# Artemisinin resistance containment project in Thailand. II: responses to mefloquine-artesunate combination therapy among falciparum malaria patients in provinces bordering Cambodia

**DOI:** 10.1186/1475-2875-11-300

**Published:** 2012-08-28

**Authors:** Wichai Satimai, Prayuth Sudathip, Saowanit Vijaykadga, Amnat Khamsiriwatchara, Surasak Sawang, Thanapon Potithavoranan, Aumnuyphan Sangvichean, Charles Delacollette, Pratap Singhasivanon, Jaranit Kaewkungwal, Saranath Lawpoolsri

**Affiliations:** 1 Bureau of Vector-borne Diseases, Department of Disease Control, Ministry of Public Health, Nonthaburi, Thailand; 2 Center of Excellence for Biomedical and Public Health Informatics (BIOPHICS), Faculty of Tropical Medicine, Mahidol University, Bangkok, Thailand; 3 World Health Organization, Mekong Malaria Programme, c/o Faculty of Tropical Medicine, Mahidol University, 420/6, Rajvithi Rd, Bangkok, 10400, Thailand; 4 Department of Tropical Hygiene, Faculty of Tropical Medicine, Mahidol University, Bangkok, Thailand

**Keywords:** Malaria containment, Artemisinin resistance, Thai-Cambodian border, Malaria surveillance

## Abstract

**Background:**

The area along the Thai-Cambodian border is considered an epicenter of anti-malarial drug resistance. Recently, parasite resistance to artemisinin-based therapies has been reported in the area. The artemisinin resistance containment project was initiated in November 2008, with the aim to limit resistant parasites and eliminate malaria in this region. This study describes the response to artemisinin-based therapy among falciparum malaria patients in the area, using data from the malaria surveillance programmed under the containment project.

**Methods:**

The study was conducted in seven provinces of Thailand along the Thai-Cambodian border. Data of *Plasmodium falciparum*-positive patients during January 2009 to December 2011 were obtained from the electronic malaria information system (eMIS) Web-based reporting system. All *P. falciparum* cases were followed for 42 days, as the routine case follow-up protocol. The demographic characteristics of the patients were described. Statistical analysis was performed to determine the cure rate of the current standard anti-malarial drug regimen--mefloquine-artesunate combination therapy (MAS). The proportion of patients who remained parasite-positive at each follow-up day was calculated. In addition, factors related to the delayed parasite clearance on day-3 post-treatment, were explored.

**Results:**

A total of 1,709 *P. falciparum*-positive cases were reported during the study period. Almost 70% of falciparum cases received MAS therapy (n = 1,174). The majority of cases were males, aged between 31 and 50 years. The overall MAS cure rate was >90% over the three-year period. Almost all patients were able to clear the parasite within 7 to 14 days post-treatment. Approximately 14% of patients undergoing MAS remained parasite-positive on day-3. Delayed parasite clearance was not significantly associated with patient gender, age, or citizenship. However, delayed parasite clearance varied across the study area.

**Conclusion:**

Anti-malarial drug-resistant parasites should be closely monitored in the area along the Thai-Cambodian border. Although the MAS cure rate in this study area was above 90%, an increasing trend of treatment failure has been reported in neighboring parts. Effective malaria surveillance is an important component to monitor drug-resistance in the malaria containment project.

## Background

Thailand and its neighboring countries have been widely known as an epicenter of drug-resistant malaria [1]. Chloroquine resistance was first reported in this area in the 1970s, followed by resistance to other anti-malarial drugs. Over the past decade, artemisinin-based therapy became the first-line protocol for the management of *Plasmodium falciparum* infections in the Greater Mekong Sub-region (GMS) [2]. The effectiveness of artemisinin-based combination therapy has been acknowledged worldwide, contributing to a reduction in the global malaria burden, especially in areas where *P. falciparum* became highly resistant to chloroquine and sulphadoxine-pyrimethamine [3]. Over the last five years, there has been increasing public health concern regarding the emergence of *P. falciparum* resistance to artemisinins along the Thai-Cambodian border, possibly spreading to other regions. Several studies have provided evidence of resistant hotspots in some western provinces of Cambodia and certain eastern provinces of Thailand, and more recently there has been serious suspicion of additional hotspots on the Thai-Myanmar border [4-6]. The artemisinin-resistance situation remains critical in areas along the Thai-Cambodian border, where the incidence of falciparum infection has been declining drastically [7].

Mefloquine-artesunate combination therapy (MAS) has been used as a first-line regimen in Thailand since 1995, in Cambodia since 2000, and in Myanmar since 2002. Results from *in-vivo* therapeutic efficacy studies conducted with MAS in the GMS between 2000 and 2010 show that MAS is still effective, with an adequate 28-day clinical and parasitological response above 90% in all sentinel sites where studies were conducted, except in some locations in Cambodia and Thailand where an increasing treatment failure rate of over 10% was observed. The treatment failure rate (PCR corrected to distinguish re-infection from recrudescence) was reaching higher levels with a 42-day follow-up protocol (20% in Cambodia and 12% in Thailand) [8]. Although the therapeutic efficacy of artemisinin-based combination therapy (ACT) has not changed dramatically, recent clinical and *in-vitro* studies have suggested that the delayed parasite clearance time may be a valid, but yet not perfect indicator of *P. falciparum* strains’ becoming less susceptible to the artemisinins, rather than a sudden change in cure rate [9]. The World Health Organization (WHO) recommended that the prevalence of patients remaining parasitaemic on day 3 (72-hours after onset of ACT) can be used as an indirect (proxy) parasitological marker of artesunate-resistant strains on the Thai-Cambodian border [10]. An increase in the proportion of patients still parasite-positive on day-3 after ACT, under strict study conditions, may indicate the emergence of suspected falciparum resistance to artemisinin derivatives in that area [8]. Median parasite clearance time can be up to 100 hours among patients with suspected artemisinin resistance, compared with less than 48 hours among patients with parasites fully susceptible to artemisinins [9].

The increasing evidence of emergent artemisinin-resistant malaria strains in the two countries has triggered regional and global attention, since resistant strains might spread worldwide, especially to other highly malaria-endemic countries in Africa, where ACT is widely used and supported by the international community [8]. The WHO, as a result, along with development partners and countries, released the Global Plan for Artemisinin Resistance Containment in 2010 aiming urgently to contain or better eliminate resistant parasites in the Greater Mekong Sub-region. If successful, the plan will prevent the further spread of artemisinin-resistant parasites to other regions and retain the gains of the previous decade’s efforts [8].

The WHO initiated the anti-malarial drug resistance containment project in Southeast Asia in November 2008, with extra funding from the Bill & Melinda Gates Foundation. The ultimate goals of the containment project were to identify and keep resistant parasites within the documented hotspot area (the Thai-Cambodian border) and ideally to eliminate *P. falciparum* malaria strains altogether, by enhancing the active, passive and individual follow-up surveillance system, and by ensuring diagnosis and full radical treatment of all confirmed malaria cases [11,12]. The Bureau of Vector-Borne Diseases, Ministry of Public Health of Thailand has implemented the containment project in seven provinces along the Thai-Cambodian border, where artemisinin resistance has been documented.

To boost the performance of the surveillance system, which was essential for the project, the electronic Malaria Information System (eMIS) was developed; its aim was to replace existing paper-based malaria reporting progressively. Web and mobile technologies were integrated into the eMIS to enhance case detection at point-of-care units, and index case investigation and active follow-up of individual patients. Paper-based data were computerized, and able to provide near real-time information that was useful at both operational and managerial levels to monitor case management in general and the occurrence of resistant parasites in targeted provinces. From data and information generated from the eMIS, this study describes the pattern of response to artemisinin-based treatment for falciparum malaria after three years of project implementation in Thailand (2009–2011), and discusses limitations of the innovative methods used.

## Methods

### Study sites

**Figure 1:**
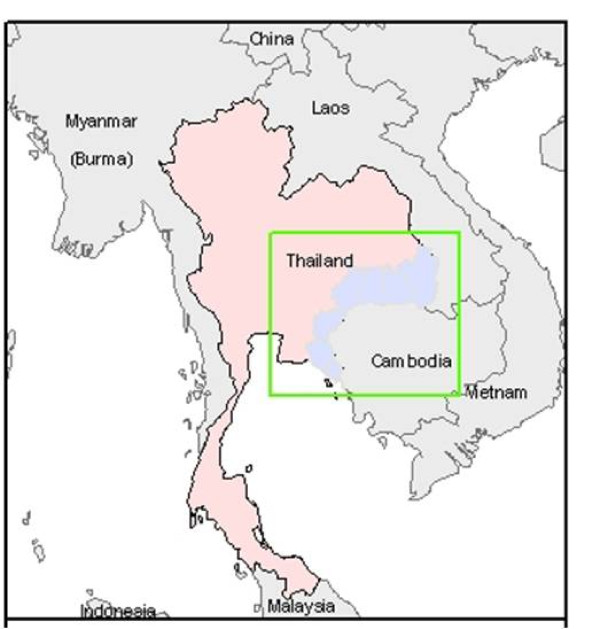
Map of study area.

Under the Thailand malaria containment project, the eMIS was developed and implemented in seven provinces along the Thai-Cambodian border (Figure 1). The study site covered those places where emergence of *P. falciparum* resistance to artemisinins has been documented (so-called zone 1), and areas where resistance has not been reported but considered at high risk due to being close to zone 1. In total, the study area covered 61 malaria posts and clinics, 27 vector-borne disease units, and 11,615 villages with 12,508 hamlets, and about seven million people. Malaria diagnosis and treatment were generally performed by malaria staff working at the malaria posts and malaria clinics, under the direct supervision of malaria teams operating at vector-borne disease units. Patients with severe malaria were referred and managed at district and referral hospitals. Rapid diagnostic tests were used for all suspected malaria cases at point-of-care units. Blood smears were also taken and sent to malaria clinics and other upper levels to confirm diagnosis. Malaria treatment was administered according to the national malaria treatment guidelines. For uncomplicated *P. falciparum* infection, three-day artesunate (total of 600 mg) and Mefloquine (total of 1,250 mg) plus primaquine (30 mg single dose) were used as the first-line drugs. However, in zone 1, where artesunate resistance has been documented, Atovaquone-proguanil (Malarone®) was recommended on direct observance (DOT) as the first-line drug to decrease the pressure on artemisinins. Routine follow-up was usually conducted for all individual malaria cases, if possible, to ensure patients’ compliance to treatment and monitor the clinical and parasitological response eight times over a period of at least 42 days.

### Data collection

Data on the *P. falciparum* cases used in this study were obtained from eMIS between January 2009 and December 2011. Since eMIS became fully functional from July 2009, malaria data reported between January and June 2009 were entered manually into eMIS. Data from eMIS were double-checked with the existing paper-based records. Details of the eMIS infrastructure are described elsewhere [13]. Briefly, the development of eMIS was based on the routine work of the malaria prevention and control programmed under the Bureau of Vector-borne Diseases (BVBD), Ministry of Public Health, Thailand. Data captured by eMIS were similar to the old paper-based reporting system, but the redundancy of the data was considerably reduced and the information workflow was better harmonized, consolidated, and more accessible across units. According to the national malaria control programmed, the management of malaria cases (diagnosis, treatment, and individual follow-up) was generally performed at malaria posts and malaria clinics at village level. Malaria case investigations were carried out for each individual patient to determine the source of infection, and the appropriateness of the measures taken. Each individual case was followed up at home by malaria staff or village malaria volunteers on days 1, 2, 3, 7, 14, 21, 28, and 42, to ensure clinical and parasitological cure. Hospitalized patients were also followed up after being discharged. Severe malaria is not common in the area, so a very small proportion of malaria patients were hospitalized. Active case detection was also performed periodically. All information gathered from both passive and active case detection, including case investigations and follow-up visits, was entered into eMIS.

Data used in the analysis included demographic data, such as gender, age, citizenship, if patients were Thai, Migrant 1 (migrants staying in the area ≥6 months), or Migrant 2 (migrants staying in the area <6 months). In addition, time (year) and place where patients received diagnosis and treatment were also used to describe the spatial and temporal distribution of cases. Locations where patients were diagnosed and treated were classified either by province or malaria-transmission area: perennial transmission in A1 villages; periodic transmission in A2; no transmission during the previous three years but still at risk of malaria epidemic due to the presence of vectors and a persistent suitable environment for malaria transmission in B1 areas; and non-transmission areas in B2 which, in principle, are not susceptible to transmission. Parasitological data on day-0 (diagnosis) and other follow-up days were categorized into either positive or negative by malaria species without counting parasites, since the data were based on actual routine day-to-day field operations. In those circumstances, with the number of records generated across provinces, especially in remote settings, it was considered difficult to cross-check the accuracy and reliability of parasite counting from all blood slides. Therefore, the data analysis does not take into account parasite-counting trends. The most common anti-malarial drug treatment promoted in the containment area was MAS (mefloquine-artesunate combination). Malarone® was strictly recommended and used in the three districts of zone 1 (in Chantaburi and Trat provinces). Recommended second-line anti-malarial drugs included quinine plus tetracycline or doxycycline.

This study used secondary data extracted from eMIS databases with no identification linked to individual patients. Official permission from the BVBD Director was received before using the data for the final analysis. The study was reviewed and approved by the Ethics Committee of the Faculty of Tropical Medicine, Mahidol University.

### Study population

**Figure 2:**
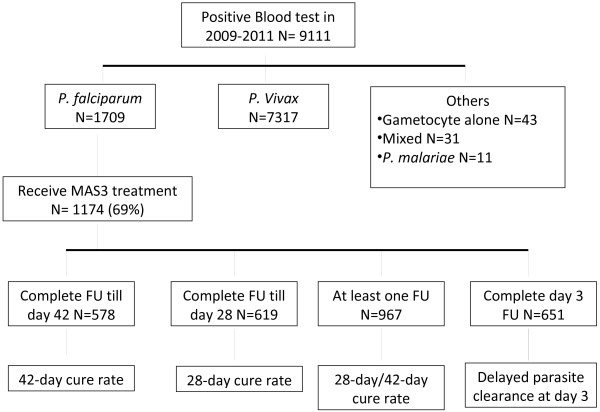
Data analysis scheme.

To determine the response pattern of *P. falciparum* to MAS, only falciparum malaria patients who received MAS treatment were included in the study. Although patient follow-up was recommended by the standard protocol, lost at follow-up was still observed, due to field constraints. The missing follow-ups may affect the assessment of MAS cure rate, therefore, analysis of patient follow-up status was performed separately, i.e. those who had fully completed parasitological follow-up through days 3, 28, and 42, and those who had at least one follow-up during the 42 days post-treatment (the ‘at least one follow-up’ group) (Figure 2). The number of patients in each group was not mutually exclusive, as patients could be classified into more than one. For example, all patients who had completed 42-day follow-up would fall into all groups (3-day, 28, 42, and one follow-up group, at least).

### Analytical methods

#### 28-day and 42-day cure rate

The response to the MAS (cure rate) was determined according to the WHO classification of responses to treatment: as early treatment failure (ETF), late clinical failure (LCF), and late parasitological failure (LPF) [10]. Only patients with adequate clinical and parasitological response (ACPR) were classified as being cured. PCR analysis is not performed to distinguish recrudescence from re-infection in a very low transmission context where re-infection is rare. The cure rates were calculated according to patient follow-up status, to avoid possible bias due to missing follow-up data. The 28-day cure rate was assessed among patients with complete 28-day follow-up, and among those patients with at least one follow-up. Similarly, the 42-day cure rate was assessed in the complete 42-day follow-up group, as well as in the ‘at least one follow-up’ group. These cure rates were calculated separately for each year during the three-year study period to determine the trend of MAS response in this area.

#### Proportion of falciparum-positive patients during 42-day follow-up

The proportion of patients who remained parasitaemic at each follow-up day until day-42 was calculated, so as to determine the actual pattern of falciparum response to MAS within 42 days after the beginning of treatment. Analysis was performed for each individual year so as to acquire a yearly pattern of falciparum response to MAS. Mean proportions of parasitaemic patients and 95% confidence intervals (CIs) were estimated, to adjust for the heterogeneity of expected “resistance level” across the seven provinces.

#### Measurement of the delayed parasite clearance time after MAS

The delayed parasite clearance time (PCT) was measured in patients who remained parasitaemic on day-3 after treatment [7,8]. Only patients who received MAS and had fully completed follow-up between day-1 and day-3 were included in the final PCT analysis. The overall proportion of patients with delayed parasite clearance was then calculated. Relative risks and 95% CIs were estimated to explore potential factors related to the delayed parasite clearance, such as basic demographic findings, years, and provinces where patients were coming from. Because the delayed parasite clearance on day-3 is considered as a proxy indicator for falciparum resistance to artemisinin, it might also be associated with the re-appearance of the parasite later on during the 42 days follow-up. Therefore, the potential association between the delayed parasite clearance on day-3 and the re-appearance of parasites between day-14 and day-42 was also explored, bearing in mind that PCR was not used to distinguish between recrudescence and re-infection.

#### Statistical analysis

The demographic characteristics of patients classified in each group were described. Crude relative risks and 95% CIs for delayed parasite clearance time were estimated, using a simple Poisson regression model (PROC GENMOD). All statistical analysis was performed using SAS version 9.2 (Center of Excellence for Biomedical and Public Health Informatics). Maps of the proportion of patients with delayed parasite clearance time were generated using ArcGIS version 10 (License:EFL647977001).

## Results

From January 2009 to December 2011, a total of 9,111 malaria cases were confirmed and reported in the seven provinces bordering Cambodia (namely Buriram, Chantaburi, Sakaew, Srisaket, Surin, Trat, and Ubon Ratchathani). Of the malaria-infected patients, 1,709 (19%) were positive for *P. falciparum*. About 69% (N = 1,174) of *P. falciparum* cases were treated with MAS, and were therefore included in the study. Among the 1,174 patients who received MAS, 967 (82%) patients had at least one follow-up between day-1 and day-42 (called ‘at least one follow-up’ group). In addition, actual numbers of patients who completed follow-up from day-1 until day-3, 28, and 42 were 651 (55%), 619 (53%), and 578 (49%) patients, respectively (Figure 2).

There was no gender difference between those with complete follow-up and those with at least one follow-up. Patients with a lack of follow-up were more likely to be young adults (aged 16–30 years), were diagnosed in 2011, were living in Srisaket Province, or were either short-term or long-term migrants and so had inconsistent migratory patterns (Table 1).

**Table 1:** **Demographic characteristic of  *Plasmodium falciparum* patients, comparing those with complete follow-up (until day-28 and day-42) and those with at least one follow-up, January 2009-December 2011**

	**Total**		**Complete 42 days FU**		**Complete 28 days FU**		**≥1 FU**	
	**N**	**%**	**N**	**%**	**N**	**%**	**N**	**%**
Total	1174	100	578	49	619	53	967	82
Gender								
Male	1067	91	520	90	561	91	880	91
Female	107	9	58	10	58	9	87	9
Age groups (years)								
0-15	73	6	37	6	40	6	59	6
16-30	416	35	176	30	193	31	343	35
31-50	538	46	276	48	294	47	437	45
50+	143	12	88	15	91	15	126	13
Fever on admission								
Yes	72	6	61	11	63	10	68	7
No	10	1	8	1	8	1	10	1
Citizen								
Thai	1024	87	529	92	568	92	863	89
Long-term Migrant	54	5	25	4	25	4	49	5
Short-term Migrant	96	8	24	4	26	4	55	6
Drug resistance area								
A1	261	22	134	23	145	23	215	22
A2	221	19	89	15	94	15	192	20
B1	178	15	87	15	94	15	137	14
B2	514	44	268	46	286	46	423	44
Year								
2009	706	60	379	66	401	65	576	60
2010	300	26	153	26	167	27	265	27
2011	168	14	46	8	51	8	126	13
Province								
Buriram	13	1	10	2	10	2	10	1
Chantaburi	40	3	13	2	13	2	17	2
Sakaew	36	3	25	4	25	4	31	3
Srisaket	387	33	110	19	114	18	297	31
Surin	158	13	85	15	107	17	126	13
Trat	164	14	75	13	77	12	152	16

Among cases with adequate follow-up, 90% were male Thai citizens. Half of patients were adults aged between 31 and 50 years, and were resident in areas classified as A1 (perennial transmission) and A2 (periodic transmission). Approximately 60% of patients were diagnosed in the first year of the containment project, in 2009. Ubon Ratchathani Province recorded the highest proportion of falciparum cases (45%), since this province is the largest and most populated (Table 1).

### Cure rate of mefloquine-artesunate combination therapy

#### 28-day cure rate

Overall, the 28-day cure rate of MAS in the area was above 90%. The decreasing trend in MAS cure rate over the three-year period was not obviously observed, i.e., the cure rate was 94% in 2001, 90% in 2010, 92% in 2009, and their 95% CIs were overlapping each other. There was no difference in cure rate among patients with complete 28-day follow-up and those with at least one follow-up (Table 2).

**Table 2:** 28-day and 42-day cure rates by year in seven provinces, January 2009-December 2011

	**2009**			**2010**			**2011**		
	**N**	**Cure**	**%Cure**	**N**	**Cure**	**%Cure**	**N**	**Cure**	**%Cure**
**28 day cure rate**									
Complete FU	401	369	92.02 (89.37-94.67)	167	151	90.42 (85.96-94.88)	51	48	94.12 (83.76-98.77)
≥1 FU	576	526	91.32 (89.02-93.62)	265	239	90.19 (86.61-93.77)	126	118	93.65 (89.39-97.91)
**42-day cure rate**									
Complete FU	379	349	92.08 (89.37-94.80)	153	142	92.81 (88.72-96.90)	46	44	95.65 (85.16-99.47)
≥1 FU	576	508	88.19 (85.56-90.83)	265	239	90.19 (86.61-93.77)	126	115	91.27 (86.34-96.20)

#### 42-day cure rate

The pattern of cure rate was similar for 28-day and 42-day follow-up. The overall 42-day cure rate remained above 90% over the whole three-year period; the year 2011 had the highest actual rate. There was no significant difference in cure rate between those with complete 42-day follow-up and those with at least one follow-up (Table 2).

#### Proportion of patients with parasites during 42-day follow-up

Among patients under MAS with adequate 42-day follow-up, parasites were cleared on day-3 in 87%, 83%, and 87% respectively in 2009, 2010 and 2011. Only a few patients (<1%) remained parasitaemic on day-7. However, parasite re-appearance during day-14 and day-42 was observed in about 5% of the patients. These patients with reappearance of the parasite were re-treated and monitored. Overall, all patients, except one in year 2009, were parasite-free on day-42 after treatment. The pattern of parasite clearance was relatively similar for the complete 42-day follow-up group and the ‘at least one follow-up’ group (Tables 3–4).

**Table 3:** Proportion of parasite-positive and 95% confidence interval (CI) by day of follow-up after anti-malarial treatment in seven provinces along the Thai-Cambodian border in complete 42-day follow-up group, January 2009-December 2011

**Day FU**	**2009**		**2010**		**2011**	
	**No. Parasite positive**	**Proportion parasite positive (95%CI)**	**No. Parasite positive**	**Proportion parasite positive (95%CI)**	**No. Parasite positive**	**Proportion parasite positive (95%CI)**
Day 0	379	100	153	100.00	46	100.00
Day 1	151	39.84 (34.91-44.77)	94	61.44 (53.73-69.15)	28	63.04 (49.09-76.99)
Day 2	104	27.44 (22.95-31.93)	51	33.33 (25.86-40.80)	12	26.09 (13.40-38.78)
Day 3	49	12.93 (9.55-16.31)	26	16.99 (11.04-22.94)	6	13.04 (3.31-22.78)
Day 7	0	-	1	0.65 (0.02-3.59)	0	-
Day 14	3	0.79 (0.16-2.30)	0	-	0	-
Day 21	6	1.58 (0.33-2.84)	1	0.65 (0.02-3.59)	0	-
Day 28	8	2.11 (0.66-3.56)	1	0.65 (0.02-3.59)	1	2.17 (0.06-6.39)
Day 42	1	0.26 (0.01-1.46)	0	-	0	-

**Table 4:** Proportion of parasite-positive and 95% confidence interval (CI) by day of follow-up after anti-malarial treatment in seven provinces along the Thai-Cambodian border in the "at least one follow-up group", January 2009-December 2011

**Day FU**	**2009**			**2010**			**2011**		
	**No. blood test**	**No. Parasite positive**	**Proportion parasite positive (95%CI)**	**No. blood test**	**No. Parasite positive**	**Proportion parasite positive (95%CI)**	**No. blood test**	**No. Parasite positive**	**Proportion parasite positive (95%CI)**
Day 0	576	576	100	265	265	100	126	126	100
Day 1	419	173	41.29 (36.57-46.00)	180	112	62.22 (55.14-69.30)	56	36	64.29 (51.74-76.84)
Day 2	420	116	27.26 (23.34-31.90)	181	63	34.81 (27.87-41.75)	56	15	26.79 (15.19-38.38)
Day 3	420	57	13.57 (10.30-16.85)	179	32	17.88 (12.26-23.49)	56	6	10.71 (2.61-18.82)
Day 7	571	0	-	264	1	0.38 (0.01-2.09)	122	0	-
Day 14	417	5	1.20 (0.39-2.78)	172	0	-	55	0	-
Day 21	415	10	2.41 (0.93-3.89)	175	3	1.71 (0.35-4.93)	55	1	1.82 (0.05-9.72)
Day 28	559	10	1.79 (0.69-2.89)	256	6	2.34 (0.49-4.20)	121	2	1.65 (0.20-5.84)
Day 42	543	1	0.18 (0.00-1.02)	243	0	-	117	0	-

**Figure 3:**
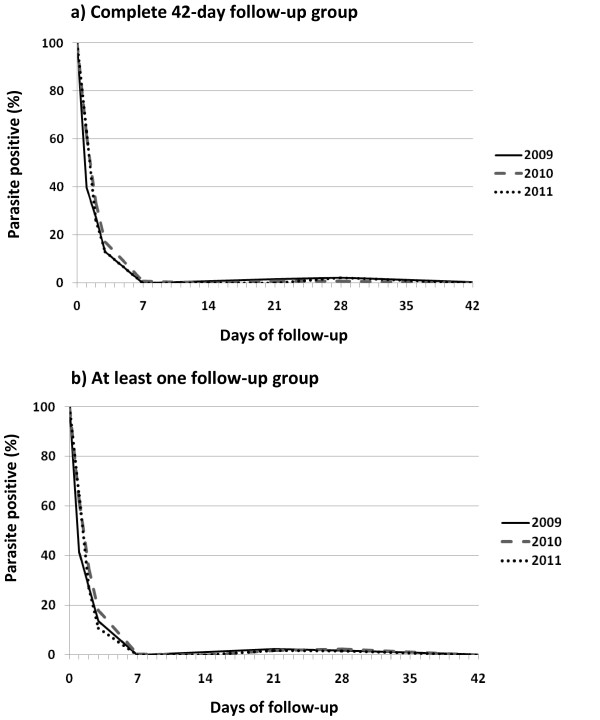
Proportion of patients parasite-positive by day of follow-up in the year 2009 (solid), year 2010 (dashed), year 2011 (dotted), for patients with complete 42-day follow-up (a) and those with at least one follow-up (b).

Figure 3 illustrates the pattern of parasite clearance during 42-day follow-up over the three-year period. Numbers of parasite-positive patients reduced rapidly during the first three days after treatment. The proportion of patients with parasitaemia almost reached zero by day-7 and slightly increased between day-14 and day-42.

#### Delayed parasite clearance time (proportion of patients still positive on day-3)

Among the 651 patients with adequate day-3 follow-up who received ACT over the three-year project period, 94 patients (14%) were still parasitaemic on day-3 (Table 5). There was no significant association between delayed parasite clearance and gender, age group, or citizenship. Patients living in seasonal transmission (A2) areas and in Srisaket Province were respectively about three and eight times more likely to have remaining parasites on day-3 as compared to patients from perennial transmission (A1) areas and other provinces. There was no significant difference in the proportion of patients with delayed parasite clearance time over the three-year project period.

**Table 5:** Relative risk and 95% confidence interval (CI) of potential risk factors for delayed parasite clearance on day-3 after artesunate-mefloquine treatment, January 2009-November 2010

	**D3 Positive**		**D3 Negative**		**RR**	**95% CI**
	**N**	**%**	**N**	**%**		
Total	94	14	557	86		
Gender						
Female	8	14	51	86	1.00	
Male	86	15	506	85	1.08	0.52-2.54
Age groups						
0-15	3	7	39	93	1.00	
16-30	31	15	176	85	2.29	0.77-9.87
31-50	48	16	258	84	2.42	0.83-10.28
50+	12	13	82	87	1.90	0.57-8.70
Citizen						
Thai	87	15	507	85	1.00	
Long-term migrant	5	17	24	83	1.21	0.40-3.02
Short-term migrant	2	7	26	93	0.45	0.07-1.54
Drug resistance area						
A1	14	9	134	91	1.00	
A2	27	27	72	73	3.59*	1.80-7.45
B1	13	12	93	88	1.34	0.59-2.99
B2	40	13	258	87	1.48	0.80-2.91
Year						
2009	56	13	361	87	1.00	
2010	32	18	146	82	1.41	0.87-2.26
2011	6	11	50	89	0.77	0.29-1.76
Province						
Buriram	0	0	10	100	-	
Chantaburi	1	7	14	93	1.00	
Sakaew	2	7	25	93	1.12	0.10-25.35
Srisaket	44	37	76	63	8.11*	1.55-149.29
Surin	22	18	99	82	3.11	0.58-57.82
Trat	15	19	66	81	3.18	0.57-59.85
Ubon Ratchathani	10	4	267	96	0.52	0.09-9.96
Reappearance						
No	86	14	530	86	1.00	
Yes	8	23	27	77	1.83	0.75-3.98

* Statistical significance.

The delayed parasite clearance time (on day-3) can be used as a proxy measurement of falciparum resistance to artemisinin derivatives. Further analysis was performed to find out if those who had a delayed parasite clearance time would be more likely to show parasite re-appearance later on during the 42-day follow-up. Results showed that only one patient tested parasite-positive on day-7, indicating that MAS cleared parasites on day-7 in almost all patients. However, some patients (n = 35, 5%) had parasites reappearing between day-14 and day-42 of treatment follow-up. Patients who had parasite reappearance after day-14 were about twice as likely to have delayed parasite clearance time, as compared to those free of parasites throughout the entire follow-up period. This association, however, was not statistically significant.

#### Spatial and temporal distribution of patients with delayed parasite clearance time

**Figure 4:**
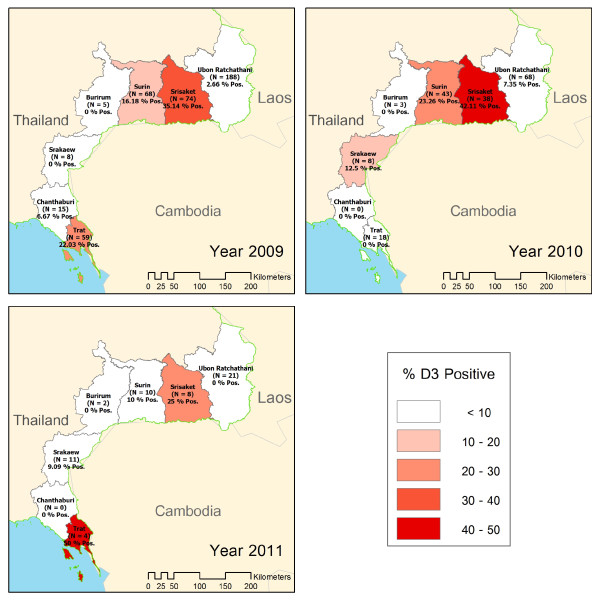
**Distribution of delayed parasite clearance on day-3 after artesunate-mefloquine treatment by province, January 2009-December 2011.** Numbers in parentheses indicate number of total blood tests per province.

The proportion of patients with delayed parasite clearance time varied across the seven provinces, showing a similar spatial distribution over the three-year period (Figure 4). Srisaket consistently reported the highest percentage of patients still parasite-positive on day-3; the worst scenario was recorded in 2010, when approximately 42% of patients were still parasitaemic on day-3.

## Discussion

The artemisinin resistance containment project started in January 2009 as the result of growing evidence of falciparum resistance to artemisinin on the Thai-Cambodian border [8]. In this area, *P. falciparum* has developed resistance to almost all anti-malarial drugs except the artemisinins [8,14]. The emergence of falciparum resistance to artemisinins would not only limit treatment options in border areas, but could compromise the management of uncomplicated malaria cases on other continents, where ACT is widely recommended and supported by international development partners. Enhancing malaria surveillance was one of the major tasks of the containment project to identify and respond to suspected artemisinin-resistant cases. The project monitored anti-malarial drug resistance, not only by strengthening the use of routine data generated from the surveillance system set up under the Thailand containment project, but also by strengthening (via extra staff and incentives) individual follow-up of all falciparum-infected patients until day-28 or 42. The total population in the seven provinces of Thailand along the Cambodian border was targeted. A three-day course of mefloquine-artesunate combination therapy (MAS) was used as first-line treatment in the containment project area, except in three districts bordering Cambodia, in Trat and Chantaburi provinces, where artemisinin resistance has been documented, leading to the choice of a non-artemisinin drug instead of MAS_3_[6].

The 28-day and 42-day cure rates of MAS observed in this study were above 90% without PCR correction. However, the situation of MAS resistance at this eastern border of Thailand was more serious, as compared to the north-western border of Thailand, where the efficacy of ACT remained above 95% [5]. There is also growing concern regarding the emergence of artemisinin resistance in an area along the Thai-Myanmar border, especially among migrant workers [15].

Parasite re-appearance until day-42 was observed in about 5% of patients. Since PCR confirmation was not performed, parasites that were reappearing in this routine monitoring study could not be distinguished either as a recrudescent infection due to drug resistance, or re-infection from new parasites. A study carried out in north-western Thailand from 1995 to 2007 reported PCR-confirmed recrudescence in less than 5% of patients under MAS [5,16]. Even if the results of this study are not confirmed by PCR, the failure rate with MAS observed during the study period remains less than 10%.

Experience over the past decades has shown that artemisinin can rapidly reduce parasite load, clearing them from blood generally after a two-day intake of treatment [17]. However, in case of suspected *P. falciparum* resistance to artemisinin, parasites are still observed in blood longer than three days after treatment, which is called “delayed parasite clearance time” [3]. Among patients managed with MAS in this study, about 30% and 15% still had parasitaemia on day-2 and day-3, respectively. Almost all patients were parasite-free on day-7, with about 2% showing parasite re-appearance during 28-day follow-up. In Pailin, western Cambodia, percentages of parasitaemic patients on day-2 and day-3 undergoing artesunate monotherapy or mefloquine-artesunate treatment were as high as 73% and 55%, respectively [7]. A recent study with high-dose artesunate monotherapy conducted in Battambang Province, western Cambodia, also showed, after treatment, a relatively high proportion of parasitaemic patients on day-2 and day-3 (48% on day-2, and 22% on day-3) [18]. The proportion of parasite-positive patients observed in the study area is relatively high compared to other locations in western Thailand and in Vietnam [5,19].

Unlike the resistance patterns of other anti-malarial drugs, the results of clinical and *in-vitro* studies indicated that artemisinin resistance is suspected by exploring parasite clearance time rather than any sudden change in therapeutic cure rate [7,9]. On the Thai-Cambodian border, the WHO recommended using the proportion of patients who remain parasitaemic on day-3 as an indicator of suspected artemisinin resistance, to be further confirmed by more sophisticated methods [10]. In this area, delayed parasite clearance on day-3 was observed in about 14% of patients. Significant factors associated with delayed parasite clearance time were people living in seasonal transmission areas or in Srisaket Province, even though the confidence intervals were quite large. The incidence of malaria in Srisaket Province was dramatically increasing during the project period, probably due to the border conflict between Thailand and Cambodia affecting containment operations, with heavy movements of non-immune soldiers. Currently, there is only one vector-borne disease unit located in Srisaket Province with limited staff and healthcare facilities. In addition to the relative inaccessibility to healthcare and malaria services in conflict zones by residents, migrants and soldiers, the overall management of malaria control operations remains difficult in this province, which might have resulted in increasing transmission and, consequently, in the spread of artemisinin-resistant parasites.

Some limitations have to be considered in this study. The pattern of parasite clearance was presented as the cumulative proportion of patients who remained parasitaemic by day after treatment. Parasite clearance curve that shows reduction of parasite load by exploring additional specific periods of time may provide more information on parasite stage-specific response to artemisinins [17]. However, the parasite clearance graph presented here should be considered as a useful tool to monitor parasite resistance in the routine surveillance system, because it is difficult to obtain accurate parasite examination and to count more often than once a day in the field situation. Study results are also subject to bias due to missing follow-up data (50%) and doubtful results, in spite of efforts made to strengthen the routine surveillance system. However, the results of this study were similar, regardless of completion of follow-up data. Additional research-based trials have to be designed to further clarify or complement results such as these. Even though the Ministry of Public Health had attempted to enforce completion and a timely analysis of data as much as possible, missing values were still observed. Approximately 50% of falciparum patients had missing parasitological data between day-1 and day-28 or day-42. The profile of patients not able to be properly followed-up was more likely to be a mobile population, such as young adults and seasonal migrant workers. However, little is known whether the resistance patterns of these two groups are different from other populations. In this study, regardless of missing data, the proportion of delayed parasite clearance on day-3 in these two groups was not significantly different from other age groups and population types. In addition, missing data were mainly (50%) observed in Srisaket Province in the conflict zone. This province was also reported to be at high risk of artemisinin resistance.

About 32% of overall falciparum malaria in this area did not receive MAS treatment. These were a combination of patients who received other second-line drugs, and others who had missing information on anti-malarial drugs received. Since there are no commercial anti-malarial drugs available in Thailand, those missing drug information were likely to have received their treatment in hospitals where standardized reporting forms were not used and computerized, as in peripheral malaria units. Unlike malaria patients who were originally detected at malaria clinics/posts under BVBD supervision, where patients’ details would be directly recorded into eMIS, details of cases at local hospitals were captured later into eMIS after admissions or treatment. Malaria treatment provided at hospital could be either first-line regimen (MAS), or second-line regimens (quinine, tetracycline or doxycycline). Further improvement in the reporting system is needed to assist data sharing between hospitals and public health sectors.

In this study, the follow-up rate on day-42 was relatively high (50%), considering constraints linked to field operation. About 90% of patients who received complete follow-up on day-7 were followed up on day-42. This allowed the study to estimate a 42-day cure rate, which is recommended to be long enough to monitor MAS resistance due to a long elimination half-life of Mefloquine [4].

## Conclusion

*Plasmodium falciparum* resistance to anti-malarial drugs has always been a major threat in the region, and globally, in malaria control and elimination [14]. In areas along the Thai-Cambodian border, resistance has been reported to almost all anti-malarial drug classes, justifying the policy shift to scale-up the use of more efficacious and expensive combinations such as ACT, to manage uncomplicated malaria infections [8,14]. Although the results of this study suggest that the therapeutic cure rate of mefloquine-artesunate combination therapy remains at an acceptable level, continuous monitoring of *P. falciparum* resistance is critical in this region.

Containment of parasites developing resistance to anti-malarial drugs is one of the major goals supported by countries and partners in the Mekong region, as well as globally, to progress from malaria control towards elimination. Malaria surveillance, with a focus on monitoring resistance, is critical to achieving such targets. The electronic Malaria Information System (eMIS) has been effectively set up and implemented to increase the performance of the existing national surveillance system. However, case detection at operational level and data sharing between units and organizations remains a day-to-day programmatic challenge, as maintaining the system requires cross-checking computerized information, then taking appropriate decisions.

## Competing interests

The authors declare that they have no competing interests. Development of the eMIS was supported by the malaria containment initiative of the WHO and the Bill & Melinda Gates Foundation.

## Authors’ contributions

SL, PS(1), WS, JK designed the study. SL, JK performed statistical analysis. PS(1), AK, SS, TP, AS worked on the design of eMIS and the applications module, and monitored and maintained the module’s implementation, and extracted data for analysis. PS(1), WS, SV were responsible for managing and supervising the overall malaria control programmer’s activities. AK, JK, DC were in charge of monitoring the progress of eMIS applications. SL, JK, WS, DC, PS(2) drafted the manuscript. All authors read and approved the final manuscript.
